# Apolipoprotein E ε4 modulates astrocyte neuronal support functions in the presence of amyloid-β

**DOI:** 10.1111/jnc.15781

**Published:** 2023-03-01

**Authors:** Rebecca M. Fleeman, Madison K. Kuhn, Dennis C. Chan, Elizabeth A. Proctor

**Affiliations:** 1 Department of Neurosurgery, Penn State College of Medicine, Hershey, Pennsylvania, USA; 2 Department of Pharmacology, Penn State College of Medicine, Hershey, Pennsylvania, USA; 3 Department of Biomedical Engineering, Pennsylvania State University, University Park, State College, Pennsylvania, USA; 4 Center for Neural Engineering, Pennsylvania State University, University Park, State College, Pennsylvania, USA; 5 Department of Engineering Science & Mechanics, Pennsylvania State University, University Park, State College, Pennsylvania, USA

**Keywords:** amyloid-β, astrocytes, ATP, cytokines, glucose, glycolysis, immunometabolism

## Abstract

Apolipoprotein E (APOE) is a lipid transporter produced predominantly by astrocytes in the brain. The ε4 variant of *APOE* (*APOE4*) is the strongest and most common genetic risk factor for Alzheimer's disease (AD). Although the molecular mechanisms of this increased risk are unclear, APOE4 is known to alter immune signaling and lipid and glucose metabolism. Astrocytes provide various forms of support to neurons, including regulating neuronal metabolism and immune responses through cytokine signaling. Changes in astrocyte function because of APOE4 may therefore decrease neuronal support, leaving neurons more vulnerable to stress and disease insults. To determine whether APOE4 alters astrocyte neuronal support functions, we measured glycolytic and oxidative metabolism of neurons treated with conditioned media from APOE4 or APOE3 (the common, risk-neutral variant) primary astrocyte cultures. We found that APOE4 neurons treated with conditioned media from resting APOE4 astrocytes had similar metabolism to APOE3 neurons treated with media from resting APOE3 astrocytes, but treatment with astrocytic conditioned media from astrocytes challenged with amyloid-β (Aβ), a key pathological protein in AD, caused APOE4 neurons to increase their basal mitochondrial and glycolytic metabolic rates more than APOE3 neurons. These changes were not because of differences in astrocytic lactate production or glucose utilization, but instead correlated with increased glycolytic ATP production and a lack of cytokine secretion in response to Aβ. Additionally, we identified that astrocytic cytokine signatures could predict basal metabolism of neurons treated with the astrocytic conditioned media. Together, these findings suggest that in the presence of Aβ, APOE4 astrocytes alter immune and metabolic functions that result in a compensatory increase in neuronal metabolic stress.

## 1 ∣ INTRODUCTION

Apolipoprotein E (APOE) is a lipid transporter found in the brain and the periphery, and is important for shuttling lipid molecules between cells to maintain membranes, modulate growth of cellular projections, and repair injured cells (Huang & Mahley, 2014; Mahley, 1988). Humans express three common APOE isoforms, APOE2, APOE3, and APOE4, which differ in only two amino acid residues (Rall et al., 1982; Zannis et al., 1981). The majority (78% of humans) express APOE3, while APOE4 is the greatest and most common genetic risk factor for Alzheimer's disease (AD) (Belloy et al., 2019; Corder et al., 1993; Farrer et al., 1997; Strittmatter & Roses, 1996). Although only 15% of people are APOE4 carriers, 60% or more of AD patients carry at least one copy of APOE4 (Farrer et al., 1997; Mahley, 2016). While the pleiotropic effects of APOE4 have been examined in multiple cell types, the mechanism by which these effects may increase AD risk is not fully understood (Belloy et al., 2019; Huang & Mahley, 2014; Perkins et al., 2016).

Astrocytes are glial cells that support neural homeostasis through regulating neuronal metabolism, maintaining synaptic connectivity, supplying neurotransmitters, and modulating immune response via cytokine signaling (Choi et al., 2014; Prebil et al., 2011; Verkhratsky et al., 2010; Verkhratsky et al., 2015). Astrocytes are the primary producers of APOE in the brain (Boyles et al., 1985). APOE shuttles cholesterol to and from neurons for maintenance of axonal growth, synaptic formation, and transport, as well as neurotransmitter release (Boyles et al., 1985; de Chaves & Narayanaswami, 2008; Huang & Mahley, 2014; Ioannou et al., 2019). APOE isoform directly affects the astrocytes that produce it, including *APOE* genotype-dependent alterations in astrocytic lipid metabolism (Sienski et al., 2021), APOE secretion (Lanfranco et al., 2021), and immune responses (De Leeuw et al., 2021). These differences remain incompletely understood, and how APOE-isoform-specific changes in metabolism and reactivity of astrocytes may negatively affect neuron health and promote disease is an open question in the field.

AD is a neurodegenerative disease characterized by two pro-teinopathies: the deposition of amyloid-β (Aβ) plaques and neurofibrillary tau tangles (Liu et al., 2013). Many healthy aging brains will accumulate Aβ and tau inclusions, thus the correlation between plaque/tangle load and neurodegeneration or cognitive impairment is only modest (Bennett et al., 2006). We hypothesize that APOE4-associated lack of astrocytic support creates vulnerabilities in neurons, leaving neurons more susceptible to stress-induced death when challenged with AD proteinopathies. Identifying the mechanisms by which APOE4 reduces astrocytic support of neurons will open new avenues of investigation in therapeutically mitigating APOE4-associated AD risk.

Here, we focus on APOE4 astrocytic and neuronal metabolism, and how Aβ stimulation of astrocytes affects the immune and metabolic support astrocytes provide neurons. We find that *APOE* genotype determines the response of astrocytes to Aβ, where APOE4 astrocytes lack an Aβ-stimulus cytokine signaling response but up-regulate glycolytic ATP production. In turn, this Aβ-stimulated APOE4 astrocytic response increases APOE4 neuronal mitochondrial and glycolytic metabolism. These findings indicate that APOE4 creates subtle shifts in reactive astrocytic responses to Aβ, which stresses neuronal metabolism, resulting in potential susceptibility to disease insults.

## 2 ∣ MATERIALS AND METHODS

### 2.1 ∣ Primary neuron and astrocyte culture

Animal protocols were approved by the Penn State College of Medicine Institutional Animal Care and Use Committee (PROTO201800531). The mice used in this study are humanized APOE knock-in animals carrying the APOE3 (B6.Cg-Apoe^em2(APOE*)Adiuj^/J, RRID:IMSR_JAX:029018) or APOE4 (B6(SJL)-Apoe^tm1.1(APOE*4)Adiuj^/J, RRID:IMSR_JAX:027894) variants (Jackson Laboratory, Bar Harbor, ME, USA). APOE4 animals were purchased as homozygotes, while APOE3 animals were purchased as heterozygotes and bred to homozygosity, with homozygosity confirmed by qPCR. Breeders were fed a standard chow diet ad libitum (Teklad 2018, Envigo). In total, 30 neonates were used. We generated primary cell cultures from postnatal day 0 or day 1 (P0/1) APOE3 and APOE4 neonates of both sexes. Each biological replicate was an individual neonate from three or more separate litters. Each neonate brain was kept separate and given its own plate wells. P0/1 neonates were sacrificed by decapitation using surgical scissors. Isolated brains were placed in cold HEPES-buffered Hanks' Balanced Salt solution (pH 7.8). The brain was dissected to isolate the cortical cap of each hemisphere, and meninges were removed.

For neuron culture, similar to validated methods (Wood et al., 2015), isolated cortices were transferred to conical tubes of warm embryonic neuron plating medium: Neurobasal Plus (Gibco), 10% fetal bovine serum (FBS; Gibco), 1x GlutaMAX (Gibco), 1x Penicillin–Streptomycin (10 000 U/mL, Gibco). Cortices were triturated in plating medium with a p1000 pipette. Cell concentration was measured using the Countess II automated cell counter (Invitrogen) and cells were plated at 15 4500 cells/well on poly-D-lysine (Gibco)-coated 24-well Seahorse plates. The cells kept at 37°C, 5% CO_2_ to allow cells to attach. 2–12 h after plating, medium was switched to Neuronal Medium: Neurobasal Plus (Gibco), 1X B27 Plus supplement (Gibco), 1X GlutaMAX (Gibco), 1X Penicillin–Streptomycin (10 000 U/mL, Gibco). Half the medium was changed every 5–7 days. Neurons were treated with astrocytic conditioned media on day 11 and assayed in Seahorse XFe24 on day 14.

For astrocyte culture, isolated cortices were transferred to conical tubes of warm glial plating medium: Dulbecco's modified eagle medium (DMEM)/F12, 20% FBS, 5 mL Penn/Strep, 5 mL 100 mM Sodium Pyruvate, 50 μL 0.1 mg/mL epidermal growth factor (EGF) stock in ddH_2_O. Cortices were triturated in plating medium with a p1000 pipette then transferred to a T-25 cell culture flask coated with Poly-D-Lysine (Gibco) with additional glial media for a total volume of 4 mL. Media were replaced every 2–3 days. Once confluent, the flask shook at 180 rpm for 2 h and media containing non-adherent glial cells was removed. Astrocytes were then washed with 1X phosphate buffered saline (PBS), removed with trypsin-ethylenediaminetetraacetic acid (EDTA), quenched with glial media, and plated in a Poly-D-Lysine coated 6-well or Seahorse plate at 1 000 000 and 50 000 cells per well, respectively. Once 75% confluent, cells were treated with corresponding conditions.

### 2.2 ∣ Amyloid-β aggregation, addition, and quantification

One milligram of human Aβ_42_ (Novex 75492034A) was solubilized in 100 μL trifluoroacetic acid (TFA) and aggregated by diluting to 22.15 μM with PBS, followed by incubation to induce aggregation at 37°C for 24 h. We added Aβ aggregates to astrocytes diluted in fresh glial media at 1 μM. After 72 h, media were removed, flash frozen in liquid nitrogen, and stored at −80°C until use.

Aβ in astrocyte and neuron media was quantified using Human Aβ42 ELISA kit (Invitrogen KHB3441), according to the manufacturer's protocol. We diluted astrocyte medium samples 4600x and neuron samples 2300x to maintain samples within the assay's sensitivity range of 15.6–1000 pg/mL.

### 2.3 ∣ Metabolic analysis

Mitochondrial and glycolytic function was analyzed with the Seahorse Cell Mito Stress Test (103015–100) and Seahorse Real-Time ATP Rate Assay (103592–100) on a Seahorse XFe24 Extracellular Flux Analyzer (Agilent). Neurons were treated on day 11 of culture with 500 μL of fresh neuronal media plus 500 μL of astrocytic conditioned media of varying treatment. This 50/50 mix of fresh neuronal medium and astrocytic conditioned medium (ACM) treatment ensured enough nutrients were provided to sustain neuronal viability. Astrocytes were treated once 75% confluent with 1 mL of media containing either 1 μM Aβ or equivalent amount of vehicle, or fresh medium controls. For both cell types, 72 h after treatment, media were removed and flash frozen in liquid nitrogen. Media were replaced with 500 μL of Seahorse assay media (Seahorse XF DMEM, 1 mM pyruvate, 2 mM glutamine, and 10 mM glucose, pH 7.4). Oxygen consumption rate (OCR) and extracellular acidification rate (ECAR) were measured at baseline and after addition of 1.5 μM oligomycin (Complex V inhibitor), 1 μM FCCP (uncoupler), and 0.5 μM rotenone/antimycin A (Complex I and Complex III inhibitors, respectively) for the Mito Stress Test, and 1.5 μM oligomycin and 0.5 μM rotenone/antimycin A for the ATP Rate Assay. Final OCR and ECAR were normalized by protein concentration (Pierce^™^ BCA Protein Assay; Thermo Scientific). Non-mitochondrial respiration was then subtracted from OCR to account for plate-to-plate variation and batch effects (Nicholas et al., 2017). All neuron Seahorse results are averages of seven to eight biological replicates (different neonates from multiple litters) and each biological replicate consisted of the median of three to six technical replicates (wells per plate). In bar plots of basal respiration, the median value of the three basal time readings was taken for each plate. All astrocyte Seahorse results are averages of three biological replicates and each biological replicate consisted of the median of three to five technical replicates.

### 2.4 ∣ Detection of glucose and lactate concentration

Glucose levels in cell culture media were measured by GlucCell Glucose Monitoring System (CESCO Bioengineering) following the manufacturer's protocol. The neuronal and glial media have glucose concentrations of 450 and 315.1 mg/dL, respectively, and the meter's range is 30–500 mg/dL. Lactate levels in cell culture media were measured by L-Lactate Assay Kit (Cayman Chemical 700510) following the manufacturer's protocol. The neuronal media do not contain lactate; the glial media do contain lactate in the mM range because of FBS. Thus, we diluted all samples 50x to maintain samples within the lactate assay's sensitivity range of 25 μM to 1 mM.

### 2.5 ∣ Cytokine concentration

We quantified the levels of 32 cytokines on the Luminex FLEXMAP 3D platform using a MILLIPLEX Mouse Cytokine/Chemokine Magnetic Bead Panel (MCYTOMAG-70 K) according to the manufacturer's protocol, accommodating to a 384-well plate format, where magnetic beads and antibodies were used at half-volume. All samples were thawed on ice and assayed in technical triplicate, with n of 9–10 samples per group.

We used an in-house automated data cleaning pipeline, available on our laboratory's GitHub page (https://github.com/elizabethproctor/Luminex-Data-Cleaning) to prepare the cytokine concentration data for analysis. Briefly, we first reviewed the bead counts of all observations, with an overall average bead count of 70 beads/well. Next, we calculated pairwise differences in fluorescence measurements within each technical triplicate. If the separation of the replicate was greater than twice the distance between the other two, it was designated an outlier and removed before further analysis. After excluding outliers (2% of measurements), we calculated the average of the remaining technical replicates for partial least squares (PLS) analysis.

### 2.6 ∣ Partial least squares discriminant analysis

We mean-centered and unit-variance scaled (i.e., Z-scored) the cytokine data prior to performing PLS analysis. For PLS discriminant analysis (PLSDA), predictors were astrocytic cytokine concentrations and outputs were the binary groupings of genotype or Aβ treatment. For PLS regression (PLSR), predictors were astrocytic cytokine concentrations, including all cytokines where >25% of samples had non-zero values, and outputs were median basal neuronal metabolism measurements from Seahorse. We performed PLS in R (v.3.6.1) using ropls (v.1.16.0) (Thévenot et al., 2015) and visualized data with ggplot2 (v.3.2.1) (Villanueva & Chen, 2019). Statistical significance for multivariate modeling was calculated by assessing accuracy through performing cross-validation with one-third of the data followed by calculating model confidence by comparing the predictive accuracy of cross-validation from our model to the distribution of cross-validation accuracies of 100 randomized models. We constructed these randomized models by randomly permuting the class assignment to the preserved X-block, conserving the data landscape to provide a valid control.

We orthogonalized each PLS model on the first latent variable (LV1), so that the parameter variance that most correlated with our chosen grouping was maximally projected onto LV1. The number of latent variables for each model was chosen based on the model with the lowest cross-validation error, and this latent variable number was maintained while constructing the distribution of randomized models for confidence calculations. Variable importance in projection (VIP) scores were calculated by quantifying the contribution of each cytokine to the prediction accuracy of our model. We calculated VIP scores by averaging the weight of each cytokine on each latent variable in the model, normalized by percent variance explained by each respective latent variable:

$${\mathrm{VIP}}_{j}=\sqrt{\frac{m}{\sum_{l=1}^{L}\mathrm{SS}(Y)}}\cdot \sum_{l=1}^{L}w_{lj}^{2}\cdot {\mathrm{SS}}_{l}(Y)$$

where m is the total number of predictors, l is the latent variable, L is the number of latent variables in the model, $w_{lj}$ is the weight (inverse loading) of predictor j on latent variable l, and ${\text{SS}}_{l}(Y)$ is the variation in class Y explained by latent variable l. Because of normalization, the average of squared VIP scores is 1, meaning that a VIP > 1 designates a parameter as having an above average contribution to our model.

### 2.7 ∣ Statistical analysis

Data are expressed as mean ± standard error of the mean, with statistical analyses conducted in Graph Pad Prism 9 (v 9.4.1). Sample size was determined with a power analysis based on previous astrocyte and neuron culture metabolic studies (Qi et al., 2021; Wu et al., 2018) to attain an effect size of 20% with *p* = 0.05 (80% power). Two-tailed t-tests were used to compare differences between neuronal basal OCR and ECAR, as well as Aβ concentration between genotypes. A two-tailed *F*-test was used to measure differences in variance between basal neuronal OCR and ECAR. We used two-way ANOVA to determine differences between basal and maximal astrocyte OCR and ECAR with two treatments, as well as astrocyte glucose and lactate in three conditions. When ANOVA results were significant, Tukey's test was utilized, which corrects for multiple comparisons using statistical hypothesis testing. We tested for outliers using the ROUT method with Q of 0.5. Statistical significance was determined using an error probability level of *p* < 0.05. All data were assessed for normality using Shapiro–Wilk test, alpha = 0.05. All data that resulted in a significant *p*-value were normal. No blinding or exclusion criteria were performed during data analysis.

## 3 ∣ RESULTS

### 3.1 ∣ Aβ-stressed APOE4 astrocytes promote increased neuronal metabolism

**FIGURE 1:**
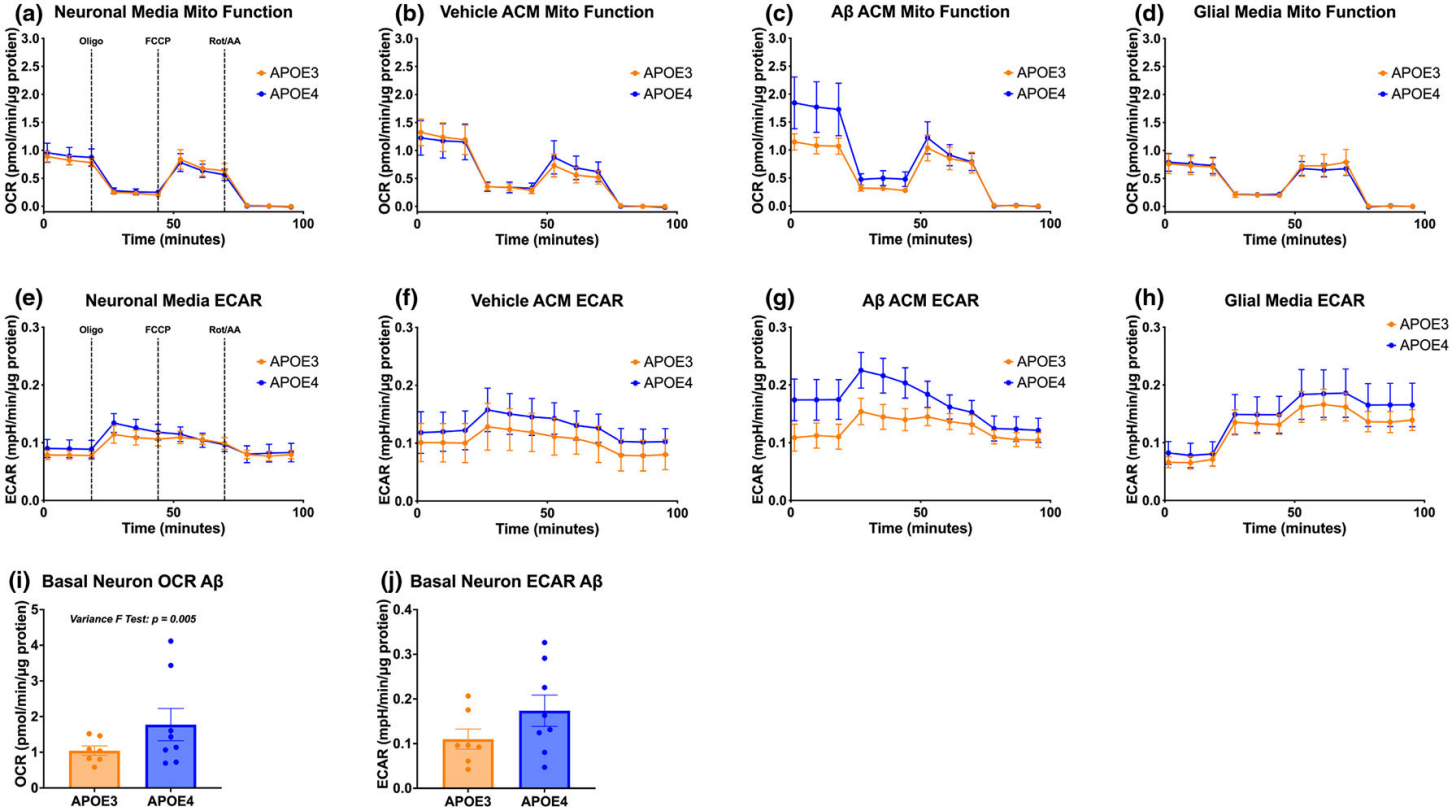
APOE4 neurons increase oxidative and glycolytic output in response to ACM from Aβ-treated astrocytes. Mitochondrial stress test oxygen consumption rates (OCRs) of APOE3 and APOE4 neurons treated for 72 hours with (a) fresh neuronal media, (b) vehicle-treated astrocyte conditioned media (ACM), (c) 1 μM Aβ-treated ACM, or (d) glial media that had not been on cells. Neurons were treated with sequential injections of mitochondrial inhibitors oligomycin A (Oligo), FCCP, and rotenone/antimycin A (Rot/AA). Corresponding extracellular acidification rates (ECARs) of mitochondrial stress tests for APOE3 and APOE4 neurons treated for 72 hours with (e) fresh neuronal media, (f) vehicle-treated ACM, (g) 1 μM Aβ-treated ACM, or (h) glial media that had not been on cells. Quantifications of basal OCR (i) and ECAR (j) of neuronal mitochondrial stress tests. N of 7 to 8 individual neonates from 3 or more separate litters per group for all experiments. (i, j) Unpaired *t*-test, two-tailed, df = 13, (i) *t* = 1.452, *p* = 0.170; (j) *t* = 1.479, *p* = 0.163.

*APOE* genotype directly affects lipid and glucose metabolism (Qi et al., 2021; Sienski et al., 2021; Wu et al., 2018). To first confirm the effects of APOE4 on neuronal mitochondrial and glycolytic metabolism, we cultured APOE3 and APOE4 primary murine neurons and quantified mitochondrial function and glycolytic rate at rest. We found no differences in the mitochondrial respiration (oxygen consumption rate, OCR) (Figure 1a) or non-mitochondrial glycolysis (extracellular acidification rate, ECAR) of neurons (Figure 1e) because of *APOE* genotype, suggesting that APOE4 does not alter neuronal resting mitochondrial respiration.

Because astrocytes are the primary producers of APOE and are uniquely situated to impact neuronal metabolism because of their support of immune, metabolic, synaptic, and trophic function, we next measured how treating neurons with astrocytic conditioned media (ACM) would affect neuronal metabolism. We cultured APOE3 and APOE4 primary murine astrocytes and treated neurons with their corresponding genotype ACM for 72 h. We again saw no change in mitochondrial respiration because of *APOE* genotype in the neurons (Figure 1b,f).

With APOE4 being the greatest genetic risk factor for AD, we next asked how stimulating astrocytes with Aβ, the primary pathological protein in AD, may cause genotype-specific changes in the factors they secrete, downstream impacting neuronal metabolism. Aβ is known to have both direct and indirect neurotoxic effects, and Aβ-specific alterations in astrocytic function have the potential to negatively impact neuronal health. We treated primary APOE3 and APOE4 astrocytes with 1 μM Aβ for 72 h and collected the ACM. Then we treated APOE3 and APOE4 primary neurons with media from Aβ-treated astrocytes (Figure 1c,g), or with glial media without cells, to rule out effects of the factors present in the glial media (Figure 1d,h). While not statistically significant, APOE4 neurons treated with APOE4 Aβ ACM exhibited 70% higher basal mitochondrial respiration (*p* = 0.170, Figure 1c,i) and 58% higher non-mitochondrial glycolytic rate (*p* = 0.163, Figure 1g,j) than APOE3 neurons treated with APOE3 Aβ ACM. However, APOE4 Aβ ACM-treated APOE4 neurons displayed significantly higher basal OCR variance (*p* = 0.005) than APOE3 Aβ ACM-treated APOE3 neurons. The combination of the biologically large difference as well as the statistically significant difference in variance led us to further explore the relationships between the differences in neuronal metabolism and astrocytic secreted factors when stressed with Aβ.

### 3.2 ∣ *APOE* genotype does not affect astrocytic lactate production or glucose utilization

**FIGURE 2:**
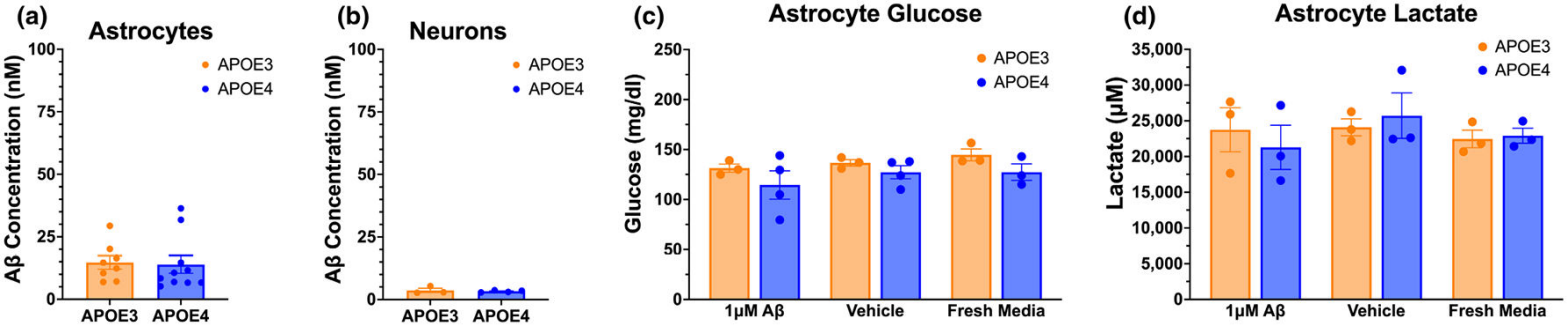
Aβ internalization, glucose consumption, and lactate production are similar in APOE3 and APOE4 astrocytes. (a) Concentration of Aβ remaining after 72-h incubation of 1 μM Aβ on astrocytes. (b) Concentration of Aβ remaining after 72-h incubation of Aβ-treated ACM on neurons. Levels of glucose (c) and lactate (d) in astrocyte media after 72-h treatment with 1 μM Aβ, vehicle, or fresh glial media. *N* of 8 to 10 individual neonates from 3 or more separate litters per group (a, b), *N* of 3 to 4 (c, d). (a, b) Unpaired t-test, two-tailed, (a) df = 16, *t* = 0.170, *p* = 0.867; (b) df = 5, *t* = 0.522, *p* = 0.624. (c, d) Two-way ANOVA; (c) df = 14, *p*_treatment_ = 0.358, *p*_genotype_ = 0.068, *p*_interaction_ = 0.885; (d) df = 12, *p*_treatment_ = 0.544, *p*_genotype_ = 0.949, *p*_interaction_ = 0.679.

To identify why APOE4 neurons treated with media from Aβ-treated astrocytes increase their basal metabolism, we first asked how much of the Aβ was left in the ACM after treatment, which would then be included in the neuron stimulation with ACM. After 72 h of incubation with 1 μM Aβ, APOE3 and APOE4 astrocytes have similar levels of Aβ left in media (14 nM, or 64 ng/mL) (Figure 2a), indicating that both APOE3 and APOE4 astrocytes internalize 99% of the Aβ used for treatment. After 1:1 dilution with fresh neuronal media to ensure adequate levels of nutrients for neuron health, 7 nM of Aβ was present in the ACM added to neuron cultures, indicating that neurons were treated with only 0.7% of a neurotoxic dose of Aβ. Following the 72-h treatment incubation, 3.5 nM of Aβ remained in the neuron culture media (Figure 2b). With no differences in Aβ levels observed in the media of APOE3 and APOE4 astrocytes or neurons, Aβ is an unlikely culprit for the observed differences in basal neuronal metabolism following exposure to ACM from Aβ-treated astrocytes.

We next asked whether Aβ-treated APOE4 astrocytes consume less glucose from the media than APOE3 astrocytes, resulting in higher levels of glucose in the ACM that might explain elevated basal metabolism in neurons. However, neither *APOE* genotype nor Aβ treatment appreciably affected glucose concentration in ACM (Figure 2c).

Finally, because astrocytes can provide lactate as a metabolic substrate to neurons, we asked whether elevated neuronal basal mitochondrial metabolism can be explained by higher lactate production by APOE4 astrocytes when treated with Aβ. However, similar to glucose, lactate levels were not altered by *APOE* genotype, nor by Aβ treatment (Figure 2d).

### 3.3 ∣ Aβ treatment increases APOE4 astrocyte glycolytic ATP production

**FIGURE 3:**
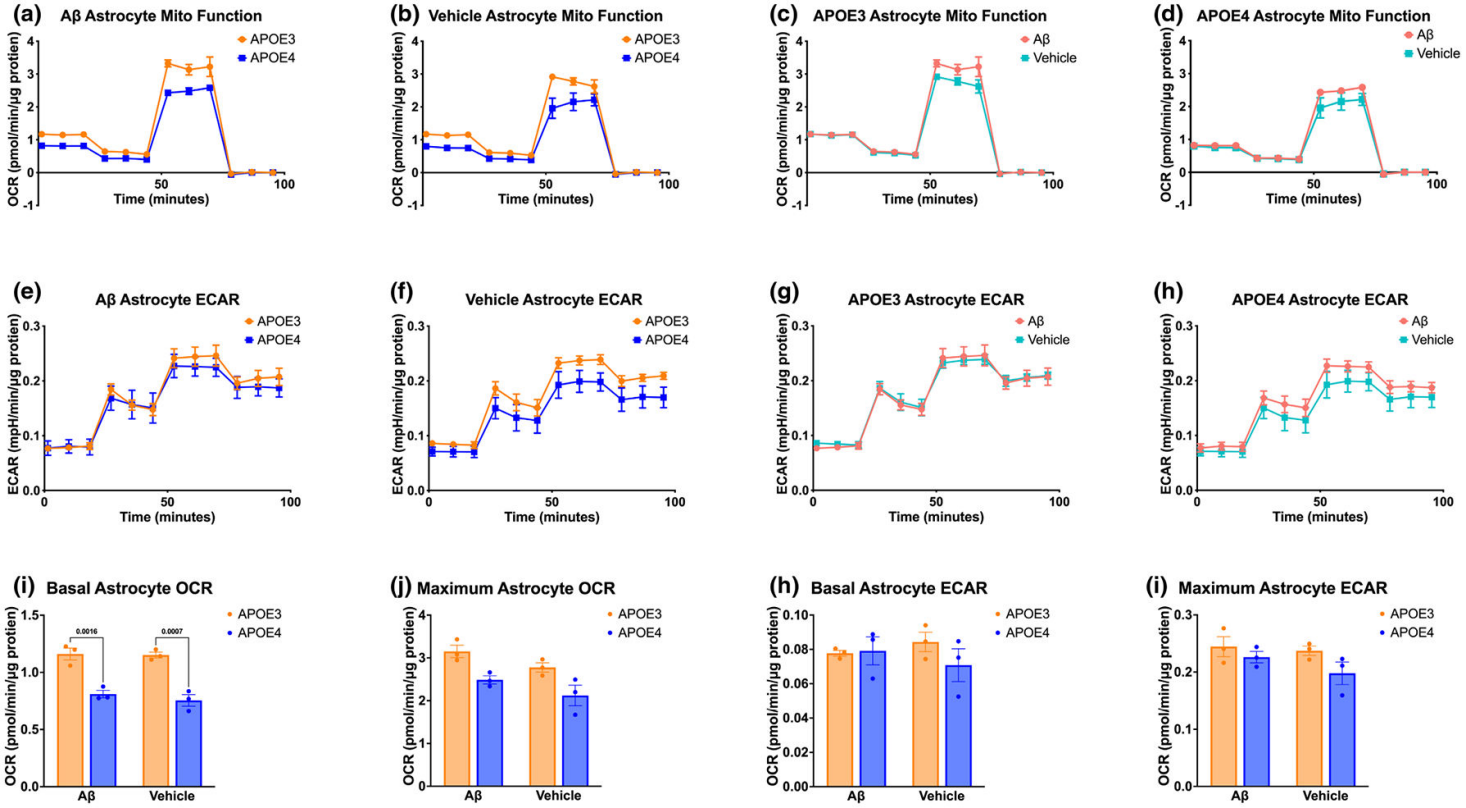
APOE4 astrocytes have lower metabolic function than APOE3 astrocytes but increased glycolytic response to Aβ. Mitochondrial stress test OCR (a, b) and ECAR (e, f) of APOE3 and APOE4 astrocytes treated for 72 h with 1 μM Aβ or vehicle. APOE3 mitochondrial stress test OCR (c) and ECAR (g) of astrocytes treated for 72 h with 1 μM Aβ or Vehicle. APOE4 mitochondrial stress test OCR (d) and ECAR (h) of astrocytes treated for 72 h with 1 μM Aβ or Vehicle. Quantifications of APOE3 and APOE4 basal and maximal OCR (i, j) and ECAR (k, l). N of 3 individual neonates per group. (i–k) Two-way ANOVA, df = 8; (i) *p*_treatment_ = 0.449, *p*_genotype_ <0.001, *p*_interaction_ = 0.602, followed by Tukey's test; (j) *p*_treatment_ = 0.047, *p*_genotype_ = 0.003, *p*_interaction_ = 0.975, followed by Tukey's test; (k) *p*_treatment_ = 0.904, *p*_genotype_ = 0.408, *p*_interaction_ = 0.314; (l) *p*_treatment_ = 0.261, *p*_genotype_ = 0.084, *p*_interaction_ = 0.491.

We quantified the mitochondrial respiration and glycolytic rates of APOE3 and APOE4 astrocytes to better understand how differences in their metabolic function may influence neuron health. APOE4 astrocytes have lower basal respiration levels in comparison to APOE3 astrocytes, both at rest (Figure 3b,i) and in the presence of Aβ (Figure 3a,i), indicating that APOE4 astrocytes operate at a lower aerobic metabolic rate than APOE3 astrocytes. Additionally, Aβ treatment did not significantly alter maximal or basal respiration, in both APOE3 and APOE4 astrocytes (Figure 3c,d,i,j). Furthermore, APOE4 astrocyte glycolytic output was not significantly different than APOE3 in resting or Aβ-treated conditions (Figure 3e-h,k-l).

**FIGURE 4:**
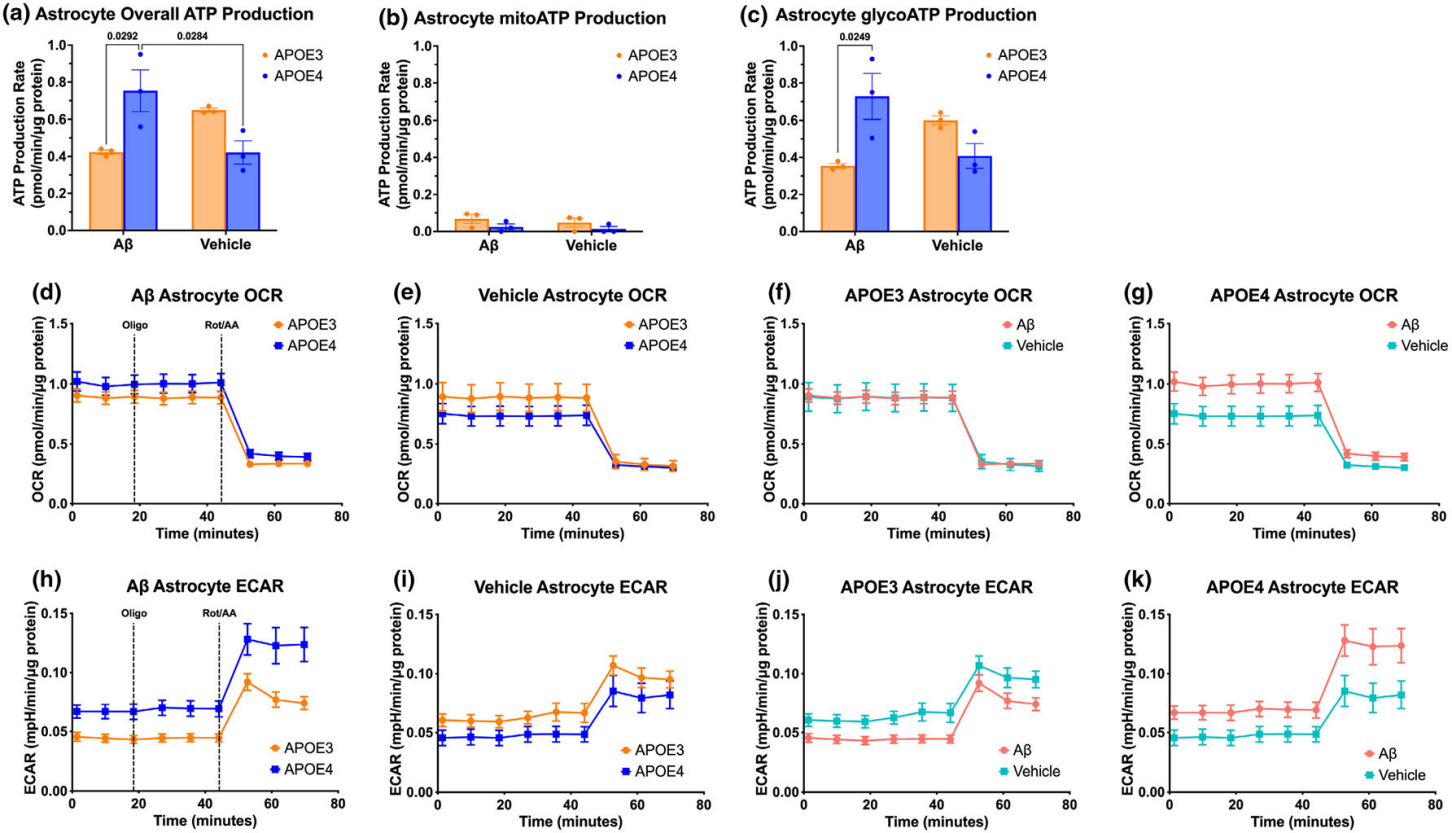
APOE4 astrocytes increase glycolytic ATP production more than APOE3 astrocytes. Total (a), mitochondrial (b), and glycolytic (c) ATP production rate comparisons between APOE3 and APOE4 astrocytes treated with 1 μM Aβ or Vehicle. OCR (d–g) and ECAR (h–k) of Seahorse ATP rate tests on APOE3 and APOE4 astrocytes treated for 72 h with 1 μM Aβ, with serial injections of mitochondrial inhibitors oligo and Rot/AA. *N* of three individual neonates per group. (a–c) Two-way ANOVA, df = 8; (a) *p*_treatment_ = 0.436, *p*_genotype_ = 0.450, *p*_interaction_ = 0.003, followed by Tukey's test; (b) *p*_treatment_ = 0.453, *p*_genotype_ = 0.087, *p*_interaction_ = 0.841; (c) *p*_treatment_ = 0.613, *p*_genotype_ = 0.238, *p*_interaction_ = 0.004, followed by Tukey's test.

We next tested whether APOE4 astrocytes may be increasing APOE4 neuronal metabolism through differences in astrocytic ATP production in the presence of Aβ, since ATP is a key signaling molecule by which astrocytes modulate neuronal activity (Boué-Grabot & Pankratov, 2017; Koizumi et al., 2005). We quantified astrocyte ATP production rates from glycolysis and mitochondrial respiration and observed that APOE4 astrocytes produced nearly double the amount of glycolytic ATP as APOE3 astrocytes in the presence of Aβ, but lower levels at rest (Figure 4a-c). Hence, APOE4 astrocytes are more metabolically reactive to Aβ than are APOE3 astrocytes.

In addition to glycolytic ATP production, APOE4 astrocytes also increased mitochondrial glucose metabolism when exposed to Aβ (Figure 4g), while APOE3 astrocytes did not (Figure 4f). However, a direct comparison of glucose metabolism between APOE3 and APOE4 astrocytes showed no significant difference in mitochondrial glucose metabolism at rest nor in the presence of Aβ (Figure 4d,e).

We next quantified the extracellular acidification rates, which reflect production of free protons from ATP and lactate in glycolysis, and CO_2_ from mitochondrial respiration. We found that APOE4 astrocytes have higher acidification rates than APOE3 astrocytes in the presence of Aβ, but not at rest (Figure 4h,i). More specifically, Aβ decreased acidification in APOE3 astrocytes but increased acidification in APOE4 astrocytes (Figure 4j,k). Based on our finding of no changes in lactate production because of *APOE* genotype or Aβ treatment in astrocytes (Figure 2d), as well as the observation that ECAR is most prominently affected by Aβ after the addition of rotenone/antimycin A (Complex I and Complex III inhibitors, respectively, that shut down the electron transport chain of oxidative phosphorylation), our data strongly support our finding that APOE4 astrocytes increase glycolytic ATP production in the presence of Aβ.

### 3.4 ∣ APOE4 astrocytes do not respond to Aβ stimulation with changes to cytokine secretion

In addition to the multitude of astrocytic metabolic pathways that impact neuronal health, cytokines secreted by glial cells also influence neuronal metabolism (Gavillet et al., 2008). APOE4 is thought to increase AD risk through several mechanisms, including by altering immune signaling networks (Martens et al., 2022). Importantly, immune signaling involves many interacting pathways, necessitating multivariate analysis to properly account for interaction and covariation of cytokine pathways. Multivariate modeling uncovers small, highly correlated differences in cytokine levels that allow us to identify immune signaling “signatures.” Using the supervised machine learning tool partial least squares (PLS), we identified patterns of astrocytic cytokine secretion associated with each *APOE* genotype.

**FIGURE 5:**
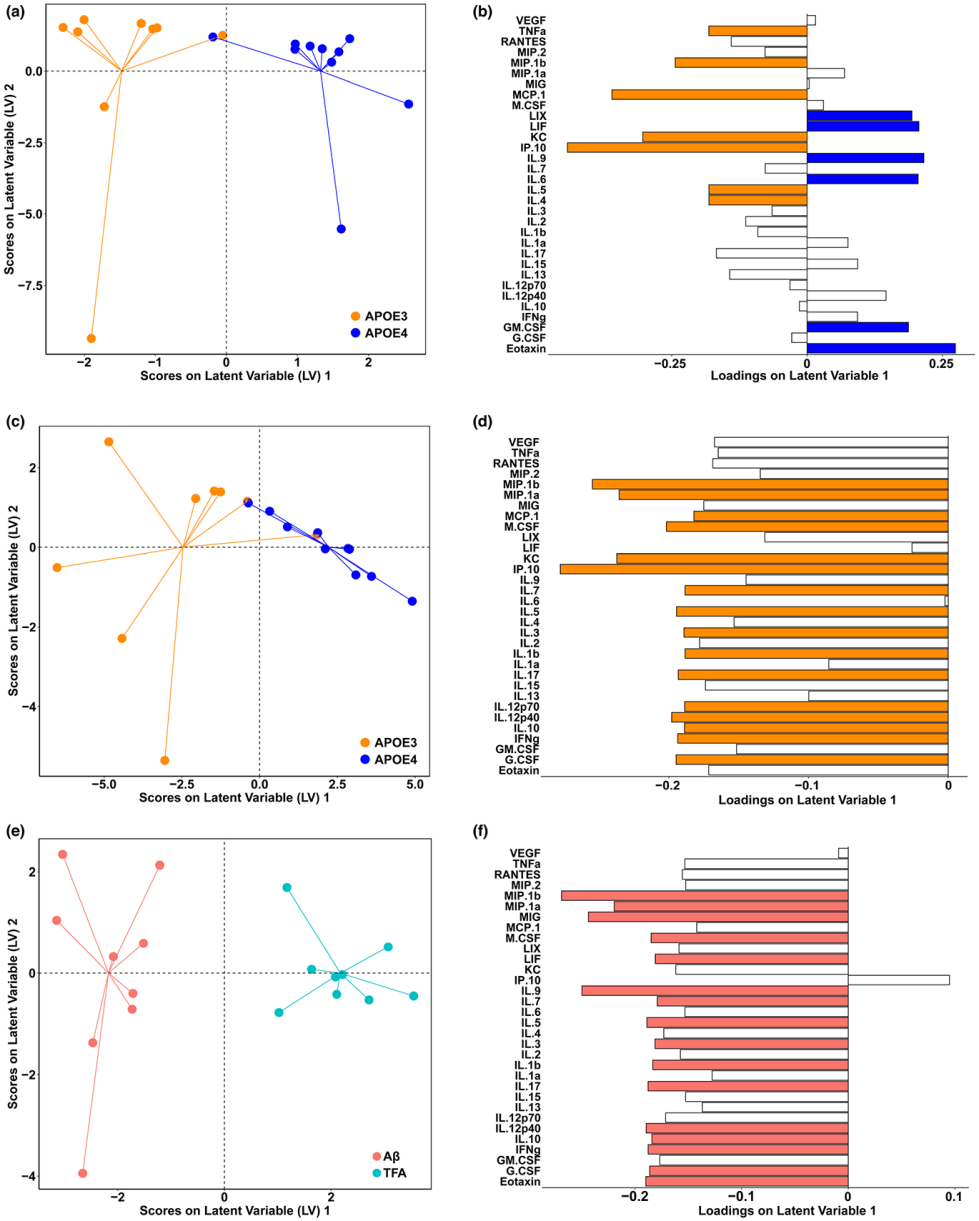
Cytokine signatures of APOE4 astrocytes demonstrate a lack of response to pathological Aβ. Partial least squares discriminant analysis (PLSDA) of vehicle-treated APOE3 and APOE4 ACM, separated by genotype (accuracy: 70.17% (5LV); confidence: 95.06%), scores plot (a) and loadings (b). Cytokines with an above average contribution to the predictive accuracy of our model, calculated by variable importance in projection scores (VIPs), are highlighted in orange. PLSDA scores (c) and loadings (d) of 1 μM Aβ-treated APOE3 and APOE4 ACM, separated by genotype (accuracy: 72.13% (2LV); confidence: 98.80%), with VIPs highlighted in orange. PLSDA scores (e) and loadings (f) of APOE3 ACM, separated by treatment with 1 μM Aβ or vehicle (accuracy: 67.22% (5LV); confidence: 96.39%), VIPs highlighted in salmon. *N* of 9 to 10 individual neonates per group. Accuracy determined by cross-validation (CV) with one-third of the data; confidence determined by comparing predictive CV accuracy of our model to the distribution of CV accuracies of 100 random models.

After quantifying the cytokine levels in conditioned media of primary APOE3 and APOE4 astrocytes using multiplexed immunoassays, we used PLS to predict *APOE* genotype based on resting levels of cytokine secretion (Figure 5a,b). *APOE* genotype has a clear effect on astrocyte immune signaling, even in the absence of AD pathology, as evidenced by the 70% cross-validation accuracy and 95% confidence in our ability to predict genotype using our model. APOE3 astrocytes secreted significantly higher levels of tumor necrosis factor α (TNFα), macrophage inflammatory protein-1β (MIP-1β), monocyte chemoattractant protein-1 (MCP-1), keratinocyte chemoattractant (KC), interferon γ-induced protein 10 (IP-10), interleukin (IL)-4, and IL-5, while APOE4 astrocytes secreted higher levels of lipopolysaccharide-induced CXC chemokine (LIX; also known as CXCL5), leukemia inhibitory factor (LIF), IL-9, IL-6, granulocyte-macrophage colony-stimulating factor (GM-CSF), and eotaxin. We note that these signatures cannot be classified as simple delineations of inflammatory or anti-inflammatory cues, but instead reveal complex differences between APOE3 and APOE4 astrocytes in the patterns of interacting immune communications.

Having discovered significant differences in cytokine secretion even at rest, we next aimed to define differences in cytokine secretion in response to an AD-relevant stimulus. When we combine all Aβ cytokine reactions for APOE3 and APOE4 astrocytes and predict genotype based on the cytokine secretion pattern of the reactive astrocytes, we observe that, similar to resting state, we are able to distinguish genotype by immune signaling reaction to Aβ stimulation (Figure 5c,d). With high accuracy (72%) and high confidence (99%), we saw that APOE3 astrocytes secrete more of all cytokines measured, consistent with our separate genotype models. VIP (variable importance in projection) cytokines which contribute greater than average to this model, helping separate APOE3 from APOE4 astrocytes treated with Aβ, include MIP-1β, MIP-1α, MCP-1, macrophage colony-stimulating factor (M-CSF), KC, IP-10, IL-7, IL-5, IL-3, IL-1β, IL-17, IL-12p70 and IL-12p40, IL-10, interferon γ (IFNγ), and granulocyte colony-stimulating factor (G-CSF). These cytokines represent a broad range of functions including inflammation, growth, tissue repair, and neuroprotection.

Focusing on the effects of Aβ stimulation in individual genotypes, we found that APOE3 astrocytes responded robustly to exposure to 1 μM Aβ, increasing levels of nearly all cytokines measured (Figure 5e,f, model cross-validation accuracy 67%, 96% confidence). Conversely, we could not distinguish the cytokine response of APOE4 astrocytes exposed to Aβ from those treated with vehicle; our PLS model performed no better than random chance (cross-validation accuracy 55%, confidence 54%). Thus, primary APOE3 astrocytes respond to Aβ by strongly up-regulating general cytokine secretion, whereas APOE4 astrocytes do not significantly alter immune signal cues in the presence of Aβ.

Overall, these three models of APOE3 and APOE4 astrocytes at rest and with Aβ stimulation, along with vehicle versus Aβ treatment in APOE3 and APOE4 astrocytes separately, demonstrate that APOE4 astrocytes have an inherent immune signaling divergence from the common APOE3 variant at rest, as well as lack of response to pathological Aβ build up in the extracellular milieu. Since Aβ stimulation did not produce predictable changes in cytokine signaling, a specific cytokine signature is not responsible for the increase in mitochondrial metabolism of APOE4 neurons.

### 3.5 ∣ Astrocytic cytokine signatures predict neuronal mitochondrial basal metabolism

**FIGURE 6:**
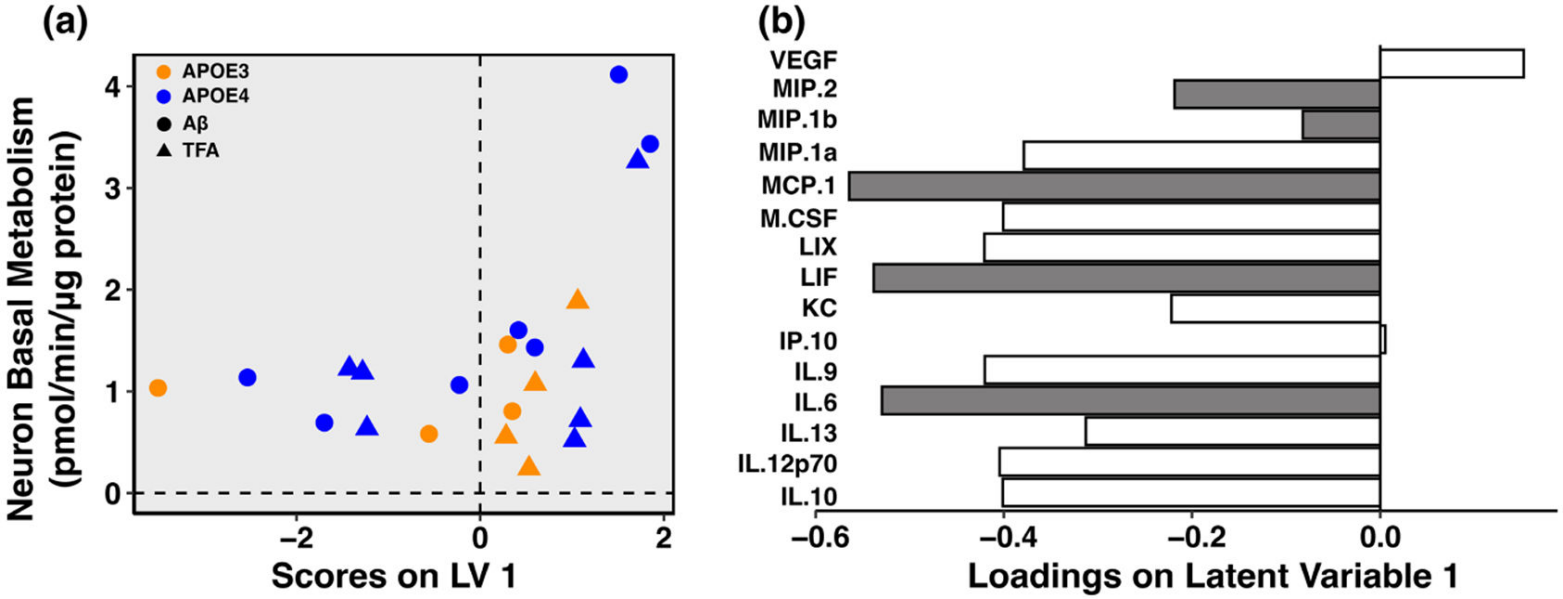
Astrocytic cytokine signatures predict basal metabolism of neurons treated with ACM. (a) Scores plot and (b) loadings for partial least squares regression (PLSR) of APOE3 and APOE4 ACM cytokine protein levels against basal neuron metabolism (as measured by oxygen consumption rate) of corresponding ACM-treated neurons (root mean squared error of cross-validation (RMSECV) of 0.765 (1LV); Confidence: 100%). Cytokines with an above average contribution to our model, calculated by variable importance in projection score, highlighted in gray; *N* = 22 biological replicates. Accuracy determined by performing cross-validation (CV) with one-third of the data; confidence determined by comparing predictive CV accuracy of our model to the distribution of CV accuracies of 100 random models.

To better identify a mechanistic relationship between astrocyte cytokine signatures and neuronal metabolism, we next performed partial least squares regression (PLSR) of the astrocytic cytokine protein levels against the resulting ACM-treated neuronal basal metabolism. We found that, with high accuracy (root mean squared error of cross-validation (RMSECV) 0.765) and 100% confidence, we are able to predict the basal metabolism of neurons with the cytokine protein levels of astrocytes of all genotypes and treatments (Figure 6a,b). Specifically, as astrocytes decreased secretion of MIP-2, MIP-1b, MCP-1, LIF, and IL-6, the corresponding neurons receiving the ACM with these cytokine signatures increased their basal mitochondrial respiration, regardless of treatment. This link further strengthens our findings that reduced cytokine secretion in APOE4 astrocytes alters neuronal metabolism.

## 4 ∣ DISCUSSION

To discover new avenues for mitigating AD risk in APOE4 carriers, we must understand how APOE4 alters responses to AD proteinopathies in a cell-type-specific manner. Astrocytes, the main producers of APOE in the brain, are crucial to supporting neuronal health and resilience, and may hold an important key to identifying mechanisms of neuron vulnerability in AD. Here, we quantified astrocytic live cell glycolytic and oxidative metabolism and immune signaling, two of their main support functions, and found that *APOE* genotype affects the astrocytic response to Aβ stimulation, which has repercussions affecting neuronal health.

We utilize one-way experiments to isolate the effects of astrocytes on neurons. While astrocyte-neuron co-culture would allow for bi-directional communication between cell types, quantification of the end-state of such cross-communication loses information on directionality of particular signals, leaving us open to misinterpretation of causality and the role of each cell type in driving particular cellular phenomena. The small amount of APOE produced by neurons (Najm et al., 2019) may affect neuronal metabolic activity in response to Aβ-treated ACM, and the size of this effect may differ between APOE genotypes. While neurons typically produce negligible amounts of APOE, they have been observed to produce APOE and APOE fragments in conditions of stress (Najm et al., 2019). Our experimental design enables us to specifically ask whether and how astrocytes are influencing markers of neuronal health.

We found that APOE4 neurons treated with ACM from Aβ-stimulated APOE4 astrocytes reacted with increased basal glycolysis and basal mitochondrial glucose metabolism, while the corresponding APOE3 neurons did not exhibit increases when treated with ACM from Aβ-treated APOE3 astrocytes (Figure 1). Without Aβ stimulation of the astrocytes, addition of ACM from APOE3 and APOE4 astrocytes to corresponding neurons evoked no change in metabolic rate. These results differ from those of Qi et al. (2021) who found that that stimulating neurons with ACM from corresponding *APOE* genotype increased maximal respiration of the neurons. While both our study and that of Qi et al. use primary cell cultures derived from the same line of APOE knock-in mice, different length of time in culture (our cells matured longer before assay) or different concentrations of drugs used in Seahorse assays (optimized differently between laboratories) may have contributed to these discrepancies in outcomes. We followed up on our findings by testing the rate of ATP production in each cell type under each condition and found that while the rate of ATP production in APOE3 neurons was higher than APOE4 neurons in all conditions (data not shown), ATP production in APOE4 astrocytes was higher than in APOE3 astrocytes when treated with Aβ (Figure 4a). Astrocyte-generated ATP can activate P2X receptors on post-synaptic neuron terminals to increase α-amino-3-hydroxy-5-methyl-4-isoxazolepropionic acid (AMPA) receptor availability, leading to increased neuronal signaling strength (Boué-Grabot & Pankratov, 2017). Thus, when we treated APOE4 neurons with media from Aβ-treated APOE4 astrocytes, higher levels of ATP in the media may have led to the rise in neuronal activity, and thus higher APOE4 neuronal metabolic output (Figure 1c). This possibility is supported by previous findings by others of a hyperactive phenotype in APOE4 neurons (Nuriel et al., 2017) that in the human brain may lead to accumulation of AD pathological proteins, resulting in the brain hypometabolism that is commonly observed in APOE4 carriers (Langbaum et al., 2010; Nuriel et al., 2017; Reiman et al., 2005).

Additionally, previous studies have investigated the differences in basal oxidative phosphorylation and glycolysis of APOE4 and APOE3 neurons and astrocytes separately. We found that APOE4 neurons have similar metabolic rates to APOE3 neurons when unstimulated (Figure 1), whereas Qi et al. (2021) and Wu et al. (2018) found that APOE4 neurons have similar basal, but lower maximal, levels of glycolysis and oxidative phosphorylation at rest. These divergences from our findings could be because of differences in optimization of drug concentrations used in Seahorse assays, as well as the types of assays performed; where we performed a mito stress test and ATP rate test, while they employed the mito stress test, glycolytic stress test, and fuel flex test. Each of these tests are valid orthogonal measurements of cellular metabolism but may elicit differing responses. We also found that APOE4 astrocytes have decreased oxidative phosphorylation and no change in glycolysis compared to APOE3 astrocytes (unless stimulated by Aβ; Figures 3 and 4), while Farmer et al. (2021) and Qi et al. (2021) found that APOE4 astrocytes decrease oxidative phosphorylation and increase glycolysis compared to APOE3 astrocytes in unstimulated conditions. In the latter study, each Seahorse result was composed of technical replicates, but no biological replicates, with brains of the same genotype homogenized together. This lack of biological replicates may decrease variability among *N*, resulting in *statistically* significant yet not *biologically* significant results. To increase experimental rigor and reproducibility, we cultured cells from individual neonate brains, with neonates from several litters generated by multiple breeder pairs, resulting in a high number of biological replicates on top of technical replicates of wells from each neonate. While this strategy increases the variability in the data, this heterogeneous population also results in increased robustness of our findings. As stated above, the results reported by Qi et al. may differ from ours because of differences in the time spent in culture or the concentrations of drugs used. We chose to assay more mature neurons based on previous observations that neurons at 11–14 days in culture produce more biologically relevant results (Seibenhener & Wooten, 2012; Wu et al., 2014).

Previous studies have quantified the metabolic effects of stimulating primary astrocyte cultures with IL-1β (Jiang & Cadenas, 2014), TNFα (Jiang & Cadenas, 2014), LPS (Robb et al., 2020), and Aβ (Allaman et al., 2010), but these studies have not accounted for the effect of APOE variants on the downstream metabolic effects of stimulus. APOE is known to modulate Aβ aggregation and clearance in a genotype-dependent manner (Holtzman et al., 2000; Liu et al., 2017), but to our knowledge, ours is the first study to examine *APOE* genotype-specific differences in astrocyte metabolic response to Aβ stimulation. Aβ has been found to increase the glucose uptake of primary astrocytes in general, demonstrating increased effects with increasing concentration, as well as with the number of hours of stimulation (Allaman et al., 2010). Along with increased glucose uptake, production of lactate and glycogen increases when astrocytes are treated with very high concentrations (25 μM) of Aβ for 48 h (Allaman et al., 2010). However, we did not observe this increase in astrocytic lactate production with a lower, more disease-physiological concentration of Aβ stimulation. The concentration of Aβ used in cell culture experiments to mimic the environment of the AD brain varies greatly and is often far beyond that found in the human brain, even in end-stage disease (LaRocca et al., 2021). The disease-physiological level of Aβ in brain tissue is cited at 50 nM, with possible localized concentrations of up to 1 μM, however, many studies use up to 50 μM of Aβ to stimulate cells (Allaman et al., 2010).

Glial activation is a key component of AD (Heneka et al., 2015) and directly effects cellular glucose and lipid metabolism (Shi et al., 2019). We sought to specifically understand the astrocytic *APOE* genotype differences in cytokine secretion upon Aβ stimulation, since astrocytes are the main producers of APOE in healthy brains and have numerous metabolically supportive functions to serve neurons, including their immune regulation support (Magistretti & Pellerin, 1999; Pellerin & Magistretti, 1994; Prebil et al., 2011; Sofroniew & Vinters, 2010). We found that even in the absence of Aβ, APOE4 primary astrocytes secrete a significantly different profile of cytokines compared to their APOE3 counterparts (Figure 5). However, in the presence of Aβ, APOE4 astrocytes did not exhibit a noticeable immune response to the stimulus, unlike the robust increase in general cytokine secretion exhibited by Aβ-treated APOE3 astrocytes. We are not the first to show lower cytokine concentrations in APOE4 astrocytes; indeed, APOE4 astrocytes stimulated with LPS were seen to have lower levels of IL-1β, IL-6, and TNFα than APOE3 astrocytes (Maezawa et al., 2006). However, we are unique in our unstimulated APOE astrocytic findings, although one study measuring resting protein levels of 10 cytokines in astrocytes concluded that APOE4 was pro-inflammatory even at baseline, but their data instead show that APOE4 astrocytes at baseline have higher levels of only TNFα, IL-8, and IL-13 while APOE3 astrocytes secreted higher levels of IL-1β (De Leeuw et al., 2021). Previous studies have evaluated differences in levels of secreted cytokines between APOE3 and APOE4 astrocytes, but their conclusions that APOE4 is more inflammatory than APOE3 are based on measurement of (a) cytokine gene expression (Shi et al., 2017) rather than protein levels; (b) only the stimulated state, without accounting for the resting state (Arnaud et al., 2022; Shi et al., 2017); or (c) very few (4 or fewer) cytokines (Arnaud et al., 2022; Lanfranco et al., 2021). As we have found here, quantification of changes in specific profiles of cytokines is more biologically relevant than the generalized and over-simplified terms of pro- or anti-inflammatory (Fleeman et al., 2022). Many studies associate *APOE4* genotype with increased inflammation (De Leeuw et al., 2021; Liu et al., 2017; Shi et al., 2017), yet the term “inflammation” may describe different phenomena in different contexts, not all of which are measured or quantified in every study. For example, tissue swelling, cellular morphological change, and molecular markers like cytokines are all used to describe an inflammatory state, but not all of these measures are necessarily in agreement in a given system. Previous work has demonstrated that APOE4 does not always increase the expression of canonically pro-inflammatory cytokines in the hippocampus, but instead has sex-based dimorphic effects in young male and female mice (Fleeman et al., 2022). Our finding that APOE4 astrocytes display a lack of change in cytokine secretion profile when stimulated with Aβ does not necessarily transfer to other measures of activation in astrocytes or other APOE4 neural cells and tissue.

In addition to inflammatory processes, cytokine signaling initiates a cascade of downstream intracellular pathways affecting a multitude of cellular processes. Thus, the lack of cytokine secretion response to Aβ we observed in A POE4 astrocytes has profound effects on the ability of cells, including neurons, in the vicinity of those astrocytes to adjust their functions in response to a potential toxic threat. For example, cytokines including TNFα and IL-1β modulate astrocytic glucose utilization, TCA cycle activity, and glycogen levels (Gavillet et al., 2008). Additionally, many cytokine signaling pathways ultimately end in modulating expression in neurons, such as glial TNFα increasing neuronal expression of AMPA receptors (Beattie et al., 2002; Stellwagen & Malenka, 2006) which modulate neuron synaptic signaling (Stellwagen & Malenka, 2006), and IL-2, IL-3, IL-4, IL-6, IL-10, and IL-12 signaling through janus kinase (*JAK*)-signal transducer and activator of transcription (*STAT*) pathways (Gadani et al., 2012; Morris et al., 2018) to increase transcription of genes, cell survival, and activation of intracellular pathways such as phosphoinositide-3-kinase (PI3K)/ protein kinase B (PKB, or Akt) and PKB/mammalian target of rapamycin (mTOR) (Gadani et al., 2012). Thus, modulation of both astrocytic cytokine secretion and metabolism may lead to our observed metabolic changes in neurons.

Recently, single-cell transcriptomic analysis has shined a spotlight on cell-type-specific contributions to AD pathology and progression (Batiuk et al., 2020; Bayraktar et al., 2020; Hasel et al., 2021; Lau et al., 2020; Mathys et al., 2019). Astrocytes in particular have been shown to have a ubiquitous role in central nervous system (CNS) diseases, which had previously been underappreciated in the field (Batiuk et al., 2020). With their important neuronal support functions, dysfunctional astrocytes contribute to a disease-promoting environment by failing to perform those functions when neurons most need them, such as in the presence of cell stressors like Aβ, leaving neurons more susceptible to injury, dysfunction, and death. Over 500 astrocytic genes are differentially expressed between AD patients and non-demented controls (Lau et al., 2020), including genes controlling neuronal synaptic signaling, which were lower in AD astrocytes, and could on a larger scale affect the coordinated firing of neural circuits affecting cognitive function (Lau et al., 2020). To our knowledge, no single-cell analysis has been done in brain tissue that identifies differences in astrocytic transcriptomes or proteomes in the context of *APOE* genotype—a gap waiting to be filled.

Overall, our study demonstrates that APOE4 astrocytes exhibit a lack of cytokine secretion response in the presence of Aβ, in tandem with increased glycolytic ATP production as compared to Aβ- exposed APOE3 astrocytes. These differing astrocyte responses to Aβ are carried into the extracellular environment and result in elevated mitochondrial and glycolytic metabolism in APOE4 neurons as compared to APOE3 neurons. This finding may potentially explain increased oxidative stress observed in the brains of human APOE4 carriers (Butterfield & Mattson, 2020; Cioffi et al., 2019; Tönnies & Trushina, 2017). Over time, elevated metabolic rate and the consequent increase in oxidative stress and free radical production can result in cellular stress and neuron death. In both healthy aging and in AD, the rates of neuron and synaptic loss increase over time (Long & Holtzman, 2019). Thus, increased oxidative stress from APOE4 astrocytes in the presence of Aβ leads to lack of neuronal support and consequent increased risk of neuron synaptic loss, contributing to a disease-promoting environment and increased risk for AD in APOE4 carriers (Corder et al., 1993; Farrer et al., 1997; Strittmatter & Roses, 1996). These findings in isolated astrocytes and neurons definitively demonstrate astrocyte-specific functional changes in the presence of Aβ, with detrimental downstream effects on neurons. More, these effects vary according to *APOE* genotype, suggesting mechanisms by which APOE4 creates a disease-promoting environment in the brain by interfering with neuronal support. Our findings highlight the need for investigations of the effects of APOE4 on inter-cell-type communications and interactions in the brain to identify new avenues for precision medicine treatment of at-risk APOE4 carriers and AD patients.

## Data Availability

The data of this study are available from the corresponding author upon reasonable request. A preprint of this article was posted on 30 September 2022 on: https://biorxiv.org/content/10.1101/2022.09.29510145v1

## Abbreviations:

ACM: astrocytic conditioned media
AD: Alzheimer's disease
AMPA: α-amino-3-hydroxy-5-methyl-4-isoxazolepropionic acid
APOE: apolipoprotein E
Aβ: amyloid-β
CNS: central nervous system
DMEM: Dulbecco's modified eagle medium
ECAR: extracellular acidification rate
EDTA: ethylenediaminetetraacetic acid
EGF: epidermal growth factor
FBS: fetal bovine serum
G-CSF: granulocyte colony-stimulating factor
GM-CSF: granulocyte-macrophage colony-stimulating factor
IFNγ: interferon γ
IL: interleukin
IP-10: interferon γ-induced protein 10
JAK: Janus kinase
KC: keratinocyte chemoattractant
LIF: leukemia inhibitory factor
LIX: lipopolysaccharide-induced CXC chemokine (also known as CXCL5)
MCP-1: monocyte chemoattractant protein-1
M-CSF: macrophage colony-stimulating factor
MIP-1β: macrophage inflammatory protein-1β
mTOR: mammalian target of rapamycin
OCR: oxygen consumption rate
PBS: phosphate buffered saline
PI3K: phosphoinositide-3-kinase
PKB: protein kinase B (also known as Akt)
PLS: partial least squares
PLSDA: partial least squares discriminant analysis
PLSR: partial least squares regression
RMSECV: root mean squared error of cross-validation
STAT: signal transducer and activator of transcription
TNFα: tumor necrosis factor α
